# Developing medical simulations for opioid overdose response training: A qualitative analysis of narratives from responders to overdoses

**DOI:** 10.1371/journal.pone.0294626

**Published:** 2024-03-28

**Authors:** G. Franklin Edwards, Cassandra Mierisch, Brock Mutcheson, Allison Strauss, Keel Coleman, Kimberly Horn, Sarah Henrickson Parker

**Affiliations:** 1 Graduate Program in Translational Biology, Medicine, and Health, Virginia Tech, Blacksburg, Virginia, United States of America; 2 Department of Basic Science Education, Virginia Tech Carilion School of Medicine, Roanoke, Virginia, United States of America; 3 Office of Assessment and Program Evaluation, Virginia Tech Carilion School of Medicine, Roanoke, Virginia, United States of America; 4 Virginia Tech Carilion School of Medicine, Roanoke, Virginia, United States of America; 5 Department of Emergency Medicine, Virginia Tech Carilion School of Medicine, Roanoke, Virginia, United States of America; 6 Department of Emergency Medicine, Carilion Clinic, Roanoke, Virginia, United States of America; 7 Department of Population Health Sciences, Virginia-Maryland College of Veterinary Medicine, Virginia Tech, Blacksburg, Virginia, United States of America; 8 Fralin Biomedical Research Institute at VTC, Roanoke, Virginia, United States of America; 9 Department of Health Systems and Implementation Science, Virginia Tech Carilion School of Medicine, Roanoke, Virginia, United States of America; Murcia University, Spain, SPAIN

## Abstract

Medical simulation offers a controlled environment for studying challenging clinical care situations that are difficult to observe directly. Overdose education and naloxone distribution (OEND) programs aim to train potential rescuers in responding to opioid overdoses, but assessing rescuer performance in real-life situations before emergency medical services arrive is exceedingly complex. There is an opportunity to incorporate individuals with firsthand experience in treating out-of-hospital overdoses into the development of simulation scenarios. Realistic overdose simulations could provide OEND programs with valuable tools to effectively teach hands-on skills and support context-sensitive training regimens. In this research, semi-structured interviews were conducted with 17 individuals experienced in responding to opioid overdoses including emergency department physicians, first responders, OEND program instructors, and peer recovery specialists. Two coders conducted qualitative content analysis using open and axial thematic coding to identify nuances associated with illicit and prescription opioid overdoses. The results are presented as narrative findings complemented by summaries of the frequency of themes across the interviews. Over 20 hours of audio recording were transcribed verbatim and then coded. During the open and axial thematic coding process several primary themes, along with subthemes, were identified, highlighting the distinctions between illicit and prescription opioid overdoses. Distinct contextual details, such as locations, clinical presentations, the environment surrounding the patient, and bystanders’ behavior, were used to create four example simulations of out-of-hospital overdoses. The narrative findings in this qualitative study offer context-sensitive information for developing out-of-hospital overdose scenarios applicable to simulation training. These insights can serve as a valuable resource, aiding instructors and researchers in systematically creating evidence-based scenarios for both training and research purposes.

## Introduction

Opioid overdose deaths continue to increase and are predominantly due to illicitly manufactured fentanyl, other synthetic opioids, and the negative impacts of COVID-19 on mental health [1, 2]. Mitigating opioid overdose deaths requires the availability of naloxone, an opioid receptor antagonist [3–6], but training bystanders to administer naloxone and treat an overdose is integral to its effectiveness [7–11]. However, there are gaps and variations in learning modalities, ranging from web-based trainings to hands-on instruction [12, 13]. Moustaqim-Barrette et al. (2021) identified these gaps as insufficient best practice guidelines applicable across jurisdictions and expressed a need for better learning strategies [14].

Edwards et al. (2023) helped close the former gap by interviewing content experts and identifying observable process measures critical to community-based opioid overdoses [15]. These nuanced hands-on resuscitation skills are designed for observation in medical simulation [12, 15]. Simulation provides a safe and controlled environment for standardized evaluations [16–18], making it an ideal method for the observation and validation of process measures. But before process measures can be observed and validated in simulation, researchers need contextual information to construct medical simulations [19, 20].

The aim of this study was to identify environmental and behavioral features of opioid overdoses for the design of medical simulations, including location, clinical symptoms, presence and behavior of bystanders, and elements of the scene. To accomplish this, the study utilized a novel source of qualitative information: the narratives of opioid overdose responders, including emergency department (ED) physicians, first responders, overdose education and naloxone distribution program (OEND) instructors, and peer recovery specialists.

## Methods

### Study site and setting

The study was conducted at a healthcare simulation laboratory located in south-central Appalachia, an area with an opioid overdose mortality rate twice as high as the national rate [21, 22]. This study was approved by the appropriate institutional review board (#19–521), and researchers obtained written or verbal consent from each participant.

### Recruitment and eligibility screening

From January 2020 to June 2020, the researchers recruited content experts from southwest Virginia using various methods such as word of mouth, emails, flyers, and an email listserv maintained by a local opioid crisis organization. Potential participants completed a screening survey via REDCap [23, 24], and those who passed were contacted for interviews.

The study aimed to recruit eligible participants with diverse backgrounds from different categories, including ED physicians, first responders, OEND instructors [25], and peer recovery specialists [26]. To be eligible, the participants must have witnessed or participated in the emergency care of at least one person who had overdosed and had naloxone administration training. The recruitment criteria of responding to at least one overdose enabled the inclusion of peer recovery specialists, who have lived experience in supporting people in recovery from substance use but may infrequently respond to clinical emergencies. OEND instructors were required to have trained at least 5 people. Each participant received a $50 compensation for their time and expertise. The researchers aimed to recruit up to 5 individuals from each category with diverse backgrounds based on previous literature.

### Semi-structured interviews

The interview guide was designed to gather comprehensive data on various aspects of opioid overdoses, including their locations, clinical presentations, the environment surrounding the patient, and bystanders’ behavior [27–30]. The interviews were conducted using open-ended questions and potential follow-ups to encourage participants to share their experiences and thoughts freely [31, 32]. The researchers made a clear distinction between illicit and prescription opioids to identify any differences in presentation and scene, without stigmatizing individuals who use or inject drugs.

A team consisting of a graduate student, a licensed physician, and a tenured researcher conducted the interviews with eligible participants either in person or via videoconference, and audio recordings were collected. To ensure accurate transcription of the interviews, an external transcription service was employed. A total of 30 transcripts were produced, with 16 pertaining to illicit opioid overdoses and 14 to prescription opioid overdoses. The transcription service utilized was https://www.thelai.com/.

The validity of the data was ensured by administering two validated surveys, the Opioid Overdose Knowledge Scale (OOKS) and the Opioid Overdose Attitude Scale (OOAS), which were slightly modified to account for intranasal administration. These surveys have been shown to be reliable and consistent in previous studies [33].

### Qualitative analysis and saturation

The 30 transcripts obtained from the interviews were coded independently by two coders, FE and AS, using NVivo 12 [34, 35]. The researchers used a qualitative content analysis approach, which involved open and axial thematic coding in two cycles [36–39]. During the first cycle of open coding, participants’ narratives were grouped according to the main questions or themes of the interviews. The second cycle of axial coding involved synthesizing subthemes from the main questions or themes. The coders worked independently and discussed their findings at three intervals. In the final cycle of coding, the researchers met to refine the themes and subthemes based on consensus and additional splitting [40, 41]. Overall, the coders identified 120 unique themes and subthemes for each section of the interview (illicit and prescription opioid overdoses). On average, each participant contributed to 40% of the total themes and subthemes, with contributions ranging from 30% to 74% [42].

The researchers aimed to capture a comprehensive understanding of the characteristics and features of out-of-hospital opioid overdoses, with a particular focus on the locations, clinical presentations, the environment surrounding the patient, and bystanders’ behavior. They acknowledged that the unique experiences and roles of each participant may have led to a more nuanced range of data than initially anticipated. Despite the small sample size, the researchers achieved saturation on the characteristics and features of out-of-hospital opioid overdoses as evidenced by the geographic location and the substantial contribution of each participant to the relevant themes and subthemes [42].

### Scenario construction

Researchers followed two criteria during scenario construction to ensure simulation scenarios were reflective of the narrative findings and summaries of the frequencies of themes across interviews. The criteria were as follows: (1) at least 40% of transcripts needed to reference a theme or subtheme or (2) it needed to be medically relevant to a clinical presentation [27, 43]. Themes and subthemes were excluded when < 40% of transcripts included them. Coders used the occurrence frequencies of the themes and subthemes across transcripts to determine which ones to include in the simulation scenarios, as this was a practical way of handling the large volumes of text and ensuring that the scenarios were reflective of the narrative findings [44].

## Results

**Fig 1:**
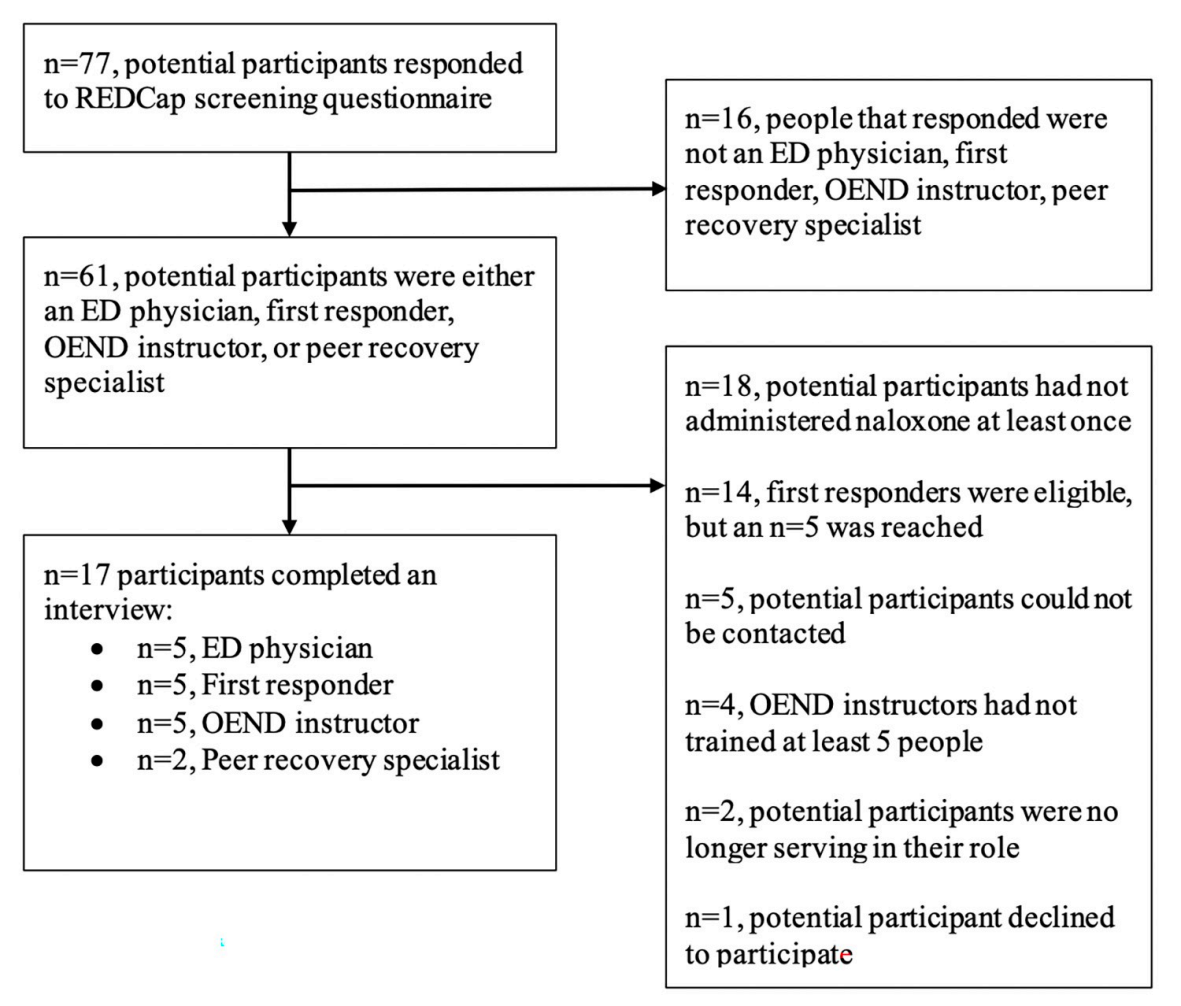
Recruitment and eligibility flowchart.

The study recruited 17 participants (Fig 1), with an equal gender distribution and all identifying as White and non-Hispanic (Table 1). The median age of the participants was 38 years old. The participants’ roles and experience varied greatly, with healthcare providers (including first responders) having the most experience. All participants had witnessed and treated at least one overdose in their lifetime, but healthcare providers (including first responders) had witnessed and treated the most (Table 2).

**Table 1:** Sample demographics.

	ED physicians (n = 4)	First responders (n = 5)	OEND instructors (n = 5)	Peer recovery specialists (n = 2)	All participants (n = 16)
Years of age, median (IQR)	42 (11)	44 (7)	37 (7.25)	36.5 (8.5)	**38 (11)**
Gender, n (%)					
Male	1 (25)	4 (80)	2 (40)	1 (50)	**8 (50)**
Female	3 (75)	1 (20)	3 (60)	1 (50)	**8 (50)**
Ethnicity, n (%)					
Non-Hispanic	4 (100)	5 (100)	5 (100)	1 (50)	**15 (94)**
No answer				1 (50)	**1 (6)**
Race, n (%)					
White	4 (100)	5 (100)	5 (100)	2 (100)	**16 (100)**
Education, n (%)					
HS diploma			2 (40)	1 (50)	**3 (19)**
Trade/vocational		1 (20)			**1 (6)**
Associate degree		1 (20)	1 (20)	1 (50)	**3 (19)**
Bachelor’s degree		2 (40)	1 (20)		**3 (19)**
Master’s Degree		1 (20)	1 (20)		**2 (13)**
Doctorate/Medical degree	4 (100)				**4 (25)**
Employment status, n (%)					
Full time	3 (75)	4 (80)	3 (60)	1 (50)	**11 (69)**
Part time	1 (25)	1 (20)	1 (20)	1 (50)	**4 (25)**
No employment			1 (20)		**1 (6)**

Three first responders had paramedic training and two first responders had advanced/mid-level (i.e., AEMT or EMT-I/EMT-Intermediate). One ED physician did not complete the demographic or overdose response surveys.

**Table 2:** Number of opioid overdoses managed and witnessed by participants.

	ED physicians (n = 4)	First responders (n = 5)	OEND instructors (n = 5)	Peer recovery specialists (n = 2)	All participants (n = 16)
Years of experience, median (IQR)	11 (3)	17 (7.5)	2.5 (1.25)	2.5 (1.5)	**8.5 (10)**
Estimate overdoses treated^a^, median (IQR)	350 (300)	175 (162.5)	1.5 (1.5)	5.5 (4.5)	**30 (246.5)**
Estimate overdoses witnessed^b^, median (IQR)	100 (1000)	127.5 (246.25)	5.5 (10)	13 (12)	**5 (61.5)**

^a^The question pertaining to the number of opioid overdoses managed by participants was worded differently based on their role: ED physicians and first responders were asked to “Estimate how many patients you have provided overdose treatment for.” OEND instructors were asked to “Estimate how many overdoses you have participated in the emergency care for.” Peer recovery specialists were asked to “Estimate how many overdoses you have provided care for.”

^b^The question pertaining to the number of opioid overdoses witnessed by participants was identical, stating: “How many overdoses have you witnessed in the community (prior to arrival at Emergency Department or never went to Emergency Department)?”

The average interview duration was 71 minutes, with a range of 56 to 88 minutes. In total, over 20 hours of audio recording were transcribed and uploaded to Nvivo12 for analysis. Researchers were mindful of the time constraints that ED physicians experience, and one physician was unable to complete the scenario characteristics section of the interview. In addition, two participants were unable to complete the prescription opioid overdose section because they had not witnessed a prescription opioid overdose.

After coders completed three cycles of coding the data was synthesized and organized according to illicit or prescription opioid overdoses (see S1 Table). S2 Table shows a complete list of themes and subthemes including those with < 40% transcript coverage (see S2 Table).

The OOKS and OOAS scores of the participants in the study were like those of people who have completed OEND training, as reported in previous literature [33]. The ED physicians had the highest OOKS scores, with a mean score of 42.00 and a standard deviation of 1.41. The first responders had the lowest OOKS scores, with a mean score of 39.40 and a standard deviation of 3.21. In terms of OOAS scores, the OEND instructors had the highest mean score of 119.40 and a standard deviation of 4.56, while the peer recovery specialists had the lowest mean score of 109.50 and a standard deviation of 13.44. The full details of the scores can be found in **S3** and **S4 Tables**.

### Illicit opioid overdoses

#### Location of overdose

The participants reported witnessing illicit opioid overdoses in various locations, including ordinary living conditions (mentioned in 56% of transcripts) that were often considered low socioeconomic households. In addition, participants reported finding people in cars (50%), hotel or motel rooms (50%), and bedrooms of either the person overdosing or a stranger’s home (50%). One ED physician who had worked as a first responder for several years described finding people in memorable locations, saying:

*Participant (ED physician)*: *We find them everywhere…it can be anything from in the bathtub packed in ice to being in bedrooms in places that people don’t know the individual that’s there*. *They’re in the back of cars*. *Usually behind stores…We have spots in the city where*…*we’ll pick five and six of ‘em up at the same time…*

#### Clinical presentation

Participants reported observing a variety of clinical presentations and body positions after an overdose. They found individuals lying on the floor or in bed (56%), sometimes in contorted positions or slumped over (44%). Physical features of the person included injection lesions in the antecubital fossa area of the arm or abscesses (75%), cyanotic lips (75%), pallor (50%), and diaphoresis (56%). An OEND instructor recounted finding someone overdosed in a car outside their workplace (a behavioral healthcare center), noting:

*Participant (OEND instructor*): *He was buckled in the seat*. *His head had slumped forward…he was doing the wheezing kind of death rattle*…*He was clearly very pale*, *and maybe starting to get a little bit of a bluish tint to his lips*…*He was definitely breaking out in a cold sweat*.

The participants reported that heroin or illicitly manufactured fentanyl overdoses can present with varying levels of consciousness (LOC) or Glasgow coma scale (GCS) scores [45], ranging from decreased LOC (GCS 8 to 10) (69%) to unresponsiveness (GCS 3) (81%). Respirations were markedly decreased (6 to 8 breaths per minute) (81%) and described as sonorous or agonal (63%). Participants with advanced medical knowledge reported the initial presence of tachycardia (130 to 140 beats per minute) that progresses to bradycardia (40 to 50 beats per minute). One first responder described the variance in LOC on presentation, stating:

*Participant (First responder*): *They literally start to nod*. *They’ll start to be off on their balance*, *and lean around*, *and lean forwards…They’re on the nod*. *They’ll be breathin’ 10*, *12 times a minute…They become a little tachycardic*, *a little pale*, *a little bit hypoxic*…*Then you get full-on respiratory depression and respiratory arrest…someone will be apneic and unresponsive*…

#### Environment surrounding the patient

Participants in the study reported finding drug paraphernalia at the scene of opioid overdoses in 94% of cases. Common items included 28-to-30-gauge syringes (69%), pill containers (56%), and white, gray, or tan powders or residue (44%), as well as other items like cotton, spoons, and tourniquets. In some cases, participants also found frozen, cold, or wet objects, which were typically found in a person’s pants as an attempt to stimulate them to counteract the overdose. As one OEND instructor explained, “*There might be syringes or residue around them*, *tourniquets*. *I’ve personally had to pull a syringe out of one person ‘cause they [overdosed]*.”

#### Bystanders’ behavior

Participants reported the presence of bystanders such as family and friends (81%) or strangers (44%). Bystander behaviors were described as fearful (75%) and distrustful of first responders and law enforcement officers (56%). Prior to administering naloxone or alerting emergency medical services (EMS) bystanders may try to stimulate persons with frozen objects or cold water. In some situations, overdose victims are placed directly in a shower or bathtub with cold water (69%). There was no clear consensus among participants if EMS are always called (63%) or whether persons are always taken to the ED. Some participants described cleaning up drug paraphernalia (25%) and fleeing the scene if EMS are called (31%). One OEND instructor reported witnessing some bystanders panic and leave while other bystanders worked together to procure naloxone, stating:

*Participant* (*OEND instructor)*: *Some of ‘em leave*. *That’s why a bunch of my friends have actually died alone*. *They weren’t even saved and [EMS] wasn’t even called ‘cause they panic and take off*. *They don’t wanna be around*. *They want their stuff*, *and they wanna go…*

According to the participants, naloxone (0.4 mg to 4 mg per mL) was administered (69%) with various routes including intranasal, intramuscular, and intravenous (63%). However, it is unclear if other clinically appropriate interventions are performed correctly or adequately. Healthcare providers often perform CPR and defibrillation with an automated external defibrillator (AED) in the event of opioid-associated cardiac arrest. According to a peer recovery specialist, bystanders are likely to administer intranasal or intramuscular naloxone, but some may be afraid to do anything about an overdose and hesitant to alert EMS for fear of getting in trouble, noting, “*…they would drop somebody off at the hospital before they would do anything*. *They don’t wanna call [EMS]*.”

### Prescription opioid overdose

#### Location of overdose

Participants reported responding to prescription opioid overdoses in personal residences in most cases (79%). Only healthcare providers, including first responders, reported responding to prescription opioid overdoses in nursing homes (29%) and assisted living facilities (21%). The types of prescription opioid overdoses were divided into unintentional overdose from a medication error (57%) and intentional suicide attempt (64%), as reported by healthcare providers.

#### Clinical presentation

Participants reported finding overdose victims in various positions, with a majority being recumbent and supine in a bed (36%). Common physical signs observed upon discovery were pallor (43%), while cyanosis (21%) and diaphoresis (14%) were less frequently reported. The pupils’ size upon discovery was unclear for most cases, with only 7% presenting with normal pupils and 21% presenting with pinpoint pupils. Unintentional or intentional prescription opioid overdoses often resulted in slightly altered LOC (GCS 11 to 12) (57%), while complete unresponsiveness was less common (43%). Decreased respiratory drive (79%) was a common finding resulting in mild bradypnea (10 to 12 breaths per minute) or moderate bradypnea (6 to 8 breaths per minute). Similar to unintended illicit overdoses, individuals initially presented with tachycardia (110 to 120 beats per minute) and hypotension (blood pressure approximately 80/60 mmHg), which progressed to bradycardia (40 to 50 beats per minute) without supplemental oxygen or overdose reversal according to participants with advanced medical knowledge.

#### Environment surrounding the patient & bystanders’ behavior

In contrast to illicit overdose, bystanders of prescription opioid overdoses will perform recommended rescue interventions including alerting EMS, performing ventilations and chest compressions, or transporting the person to an ED (50%). Bystanders at the scene usually include family and friends (86%) who display concern for the person (50%) and are supportive or helpful to EMS (21%). Participants described finding empty pill bottles (64%) and medication lists (21%). One first responder described arriving on scene after a family member had administered naloxone to their spouse, stating:

*Participant (First responder)*: *…when you come in and you see an older man laying there in bed*, *that’s the last thing you’re thinkin*’, *is overdose*. *They’re sayin’*, *“Well*, *he’s not acting right*. *He’s kinda lethargic*,*” so you’re thinkin’*, *oh*, *well is it a stroke*? *Is it a heart attack*? *“Well*, *what medications is he on*?*” Then they start tellin’ you and like*, *“Well*, *is there any chance he took*, *like*, *two of these instead of one*?*”*

Based on the information provided, it appears that participants with advanced medical knowledge have different strategies for managing prescription opioid overdoses, which depend on the person’s level of consciousness and the type of opioid consumed. Surprisingly, 43% of the participants did not administer naloxone. It is possible that these participants have alternative treatment approaches that they consider more suitable for certain situations or that other factors, such as concerns about potential adverse effects, may have influenced their decision not to use naloxone.

## Discussion

The current study provides data regarding characteristics of illicit and prescription opioid overdose scenes from the points of view of a variety of observers, allowing the development of effective medical simulations for various groups [12, 15, 46]. The narratives and experiences shared by opioid overdose responders in this study provide valuable context and details for developing medical simulations authentic to people who use opioids (PWUO). The diverse groups of participants, including individuals with personal histories of opioid use, offer crucial perspectives for future research in this field. However, it is important to note that personal information disclosure was not systematically collected to safeguard participants’ privacy and confidentiality.

The utilization of simulation methodologies to study opioid overdose response is a relatively recent development, spanning just the last decade [12, 15]. As identified by Edwards et al. (2020), at least nine studies have varied in their use of simulation modalities, the level of fidelity, and the rationale behind their contextual elements (Table 3). Notably, none of the high-fidelity simulation studies identified in Edwards et al. (2020) provide detailed insights into how they developed their simulation designs. It is evident that these simulation studies may not provide sufficient detail about their simulation methods, limiting their generalizability and making it challenging for others to replicate.

**Table 3:** Opioid overdose simulation scenario descriptions in the current literature.

Authors	Year	Description of simulation
McDermott and Collins	2012	Low fidelity setting: manikin located on a table
Edwards et al.	2015	High fidelity setting: simulated a home environment, manikin located on a couch, introduced distractions (e.g., TV)
Kim et al.	2016	High fidelity setting: simulated an emergency room environment, manikin located in a hospital bed
Krieter et al.	2016	High fidelity setting: simulated a home environment, manikin located on the floor, introduced distractions (e.g., TV and radio playing)
Kobayashi et al.	2017	High fidelity setting: simulated a public environment, manikin located on the floor, introduced distractions (e.g., street noise recordings of an approaching police car)
Eggleston et al.	2018	Low fidelity setting: simulated a public environment, manikin located on a table, introduced distractions (e.g., surrounding spectators)
Goldberg et al.	2018	High fidelity setting: simulated a public environment, manikin located on a sidewalk, introduced distractions (e.g., surrounding spectators)
Eggleston et al.	2019	Low fidelity setting: simulated a public environment, manikin located on a table, introduced distractions (e.g., surrounding spectators)
Franko et al.	2019	High fidelity setting: simulated a home environment, patient actor located on the floor, introduced distractions (e.g., panicked bystander)

Adapted from Edwards et al. (2020). See S1 File for a list of works cited in Table 3.

Other researchers are actively employing simulation methodologies for opioid overdose response training and have published research on its mutually beneficial outcomes [46, 47]. However, no prior studies have offered detailed and specific observations as evidence for deriving key elements in the creation of medical simulations. Given the recent surge in interest and application of medical simulations for training non-clinical personnel, this research is highly relevant at this time [48–50]. To the best of the authors’ knowledge, this study represents the first attempt to develop medical simulations for opioid overdose response training by drawing insights from individuals who have firsthand experience in responding to overdoses.

While the existing literature provides information on factors such as gender [51, 52], socioeconomic status [53], physical or mental comorbidities [54–56], urban and rural locations [53], distance to the nearest naloxone distribution site [57], common overdose settings [58], intubation [59], and the administration of naloxone [59], it frequently lacks in-depth contextual information. There is limited research on the locational and contextual aspects of opioid overdoses; therefore, narratives centered on responders’ experiences are particularly valuable for developing simulations [58, 59]. The findings of this study complement the existing literature and contribute to the development of more effective and contextually relevant medical simulations for opioid overdose response training.

According to Treitler et al. (2021), personal residences are the most common location for opioid overdoses, while unstably housed individuals tend to overdose more frequently in non-residential settings [58]. Their study findings highlight the need for personalized and context-specific approaches for preventing opioid overdoses. In response to this, the authors developed four simulation scenarios that are set in personal residences or cars, each representing a unique clinical presentation, bystander behavior, and/or presence of drug paraphernalia. The scenarios, which are detailed in S5 Table, are designed for a single rescuer who is the first to arrive at the scene of a community-based overdose scenario but can also be adapted for use with multiple rescuers. According to participants, multiple bystanders are often present during opioid overdoses, which presents an opportunity for further research on team dynamics among PWUO, including their impact on EMS activation, naloxone administration, rescue breathing, chest compressions, and the use of AEDs. The scenarios are designed to be useful for all rescuers, regardless of their level of training and experience.

Regarding clinical presentation, Banta-Green et al. (2017) conducted a study and found that heroin overdose victims are typically younger and male, have miotic pupils, are less likely to be intubated, and more likely to receive naloxone compared to prescription opioid overdose victims [59]. Although there were no significant differences in initial respiratory rate and GCS score between heroin and prescription opioid overdose victims, the qualitative findings from the current study suggested that there may be a difference in the severity and duration of overdose between the two groups. These qualitative findings were used to inform the development of medical simulations for opioid overdose response training, with contextual information organized around two conditions: one for a person in respiratory depression requiring one or more doses of naloxone, and another for a person experiencing an opioid-associated cardiac arrest requiring ventilations, chest compressions, and defibrillation. More information on these contextual details can be found in S5 Table.

It is interesting to note that Whittall et al. (2022) found that PWUO view simulation as a valuable opportunity to gain practical experience and improve their self-efficacy in responding to opioid overdoses [46]. This suggests that involving PWUO in the development and implementation of opioid overdose response simulations could be beneficial in promoting their engagement and effectiveness. The authors also highlight the potential benefits of low-fidelity simulations, which are more accessible and feasible in low-resource settings [60, 61], and can offer important learning opportunities that can be adapted to suit the needs of different communities [62]. Bringing simulation education to community settings, such as shelters or harm reduction facilities, could increase accessibility and help to address the unique challenges of overdose response in these settings [46, 63, 64]. Overall, incorporating diverse perspectives and considering the practical constraints of different settings is important in developing effective and accessible simulation-based training programs for opioid overdose response.

It is important to note that the use of non-recommended rescue interventions can be detrimental to the health of the individual experiencing an opioid overdose. One common theme observed in scenes of illicit opioid overdose was the use of wet or frozen objects in clothing or individuals submerged in cold water, which lacks any proven clinical benefit and may delay effective treatment [65–67]. The participants’ recognition of the significance of non-recommended rescue interventions highlights the need for effective education and training in opioid overdose response, particularly in teaching clinically meaningful hands-on resuscitation skills and discouraging the use of such interventions. By using simulation as an educational tool, potential harmful strategies can be identified and discouraged, while promoting effective and evidence-based interventions. This can ultimately lead to better outcomes for individuals experiencing opioid overdoses.

The study has several limitations that must be considered when interpreting the results. First, the small sample size and the ethnically and racially homogeneous composition of the participants limit the generalizability of the findings. Additionally, the under-representation of peer recovery specialists may have limited the perspectives included in the study. Second, the study was conducted in southwest Virginia, and the experiences and perspectives of individuals involved in opioid overdose response in other regions or countries may differ. Third, as with any qualitative analysis, some of the richness of the data may have been lost during the coding process, and there may have been biases introduced during the development of codes, themes/subthemes, and medical simulations. However, the authors took steps to limit bias through iterative cycles of coding and discussion, review of published guidelines, and feedback from board-certified physicians. Fourth, the study did not explore potential differences in opioid overdose response among different racial or ethnic groups. Fifth, the study relied on self-report and retrospective recall, which may be subject to recall bias. Finally, the study did not include a comparison group, which limits the ability to determine if the experiences and perspectives of the participants are unique to this population. Despite these limitations, the study provides important insights into the experiences and perspectives of individuals involved in opioid overdose response and highlights the need for tailored and contextualized training in this area.

## Conclusion

The study provides valuable insights into the experiences and perspectives of individuals involved in opioid overdose response. The authors recommend that future research should focus on the development and evaluation of medical simulations for out-of-hospital opioid overdose response training, which can help teach clinically meaningful hands-on resuscitation skills and discourage the use of non-recommended rescue interventions, including those used by PWUO. The authors also suggest that future studies should aim to recruit a more diverse sample of participants and explore potential differences in opioid overdose response among different racial or ethnic groups. Furthermore, the effectiveness of specific interventions or strategies for opioid overdose response should be investigated in future research. Overall, the study underscores the need for tailored and context-specific training for opioid overdose response to improve outcomes for those affected by opioid use disorder.

## Supporting information

S1 Table Themes and subthemes (at least 40% transcript coverage) of the locations and physical characteristics of heroin and illicitly manufactured fentanyl, and prescription opioid overdoses as described by participants and organized by coders. ED; emergency department, FR; first responder, OEND: OEND instructor, PRS; peer recovery specialist. ^a^ ‘Bystanders’ ‘Affects’ ‘Distrust’ include 1 transcript from the prescription opioids section because participants referenced illicit opioids. ^b^ ‘Bystanders’ ‘Behaviors’ include 2 transcripts from the prescription opioids section because participants referenced illicit opioids. ^c^ ‘Not recommended rescue interventions’ include 1 transcript from the prescription opioids section because participants referenced illicit opioids.
(DOCX)

S2 Table All themes and subthemes of the locations, physical characteristics, bystanders’ behaviors, and items near the persons of an illicit or prescription opioid overdose as described by participants and organized by coders. ED; emergency department, FR; first responder, OEND: OEND instructor, PRS; peer recovery specialist. ^a^ ‘Bystanders’ ‘Affects’ ‘Distrust’ include 1 transcript from the prescription opioids section because participants referenced illicit opioids. ^b^ ‘Bystanders’ ‘Behaviors’ include 2 transcripts from the prescription opioids section because participants referenced illicit opioids. ^c^ ‘Clean up drug paraphernalia’ include 1 transcript from the prescription opioids section because participants referenced illicit opioids. ^d^ ‘Not recommended rescue interventions’ include 1 transcript from the prescription opioids section because participants referenced illicit opioids.
(DOCX)

S3 Table Average opioid overdose knowledge scale (OOKS) scores among participants. One ED physician did not complete the OOKS.
(DOCX)

S4 Table Average opioid overdose attitude scale (OOAS) scores among participants. One ED physician did not complete the OOAS.
(DOCX)

S5 Table Four medical simulations for out-of-hospital opioid overdoses including examples of illicit and prescription opioids. (DOCX)

S1 File References in Table 3. (DOCX)
