# Supplementary material for: Developing medical simulations for opioid overdose response training: A qualitative analysis of narratives from responders to overdoses
Source: PLoS One. 2024 Mar 28;19(3):e0294626. doi: 10.1371/journal.pone.0294626 (PMC10977769; doi:10.1371/journal.pone.0294626)
Supplement: S1 Table — ED; emergency department, FR; first responder, OEND: OEND instructor, PRS; peer recovery specialist. a ‘Bystanders’ ‘Affects’ ‘Distrust’ include 1 transcript from the prescription opioids section because participants referenced illicit opioids. b ‘Bystanders’ ‘Behaviors’ include 2 transcripts from the prescription opioids section because participants referenced illicit opioids. c ‘Not recommended rescue interventions’ include 1 transcript from the prescription opioids section because participants referenced illicit opioids. (DOCX) [file pone.0294626.s001.docx]

**S1 Table.** Themes and subthemes (at least 40% transcript coverage) of the locations and physical characteristics of heroin and illicitly manufactured fentanyl, and prescription opioid overdoses as described by participants and organized by coders

| **Illicit opioids** | | **# of transcripts** | **% of transcripts** | **Expert** | **Prescription opioids** | | **# of transcripts** | **% of transcripts** | **Expert** |
| --- | --- | --- | --- | --- | --- | --- | --- | --- | --- |
| **Location** | |  |  |  | **Location** | |  |  |  |
| 1. Car | | 8 / 16 | 50% | ED  FR  OEND | 1. Personal residence | | 11 / 14 | 78.57% | All |
| 1. Hotel or motel | | 8 | 50% | All |  |  |  |  |  |
| 1. Personal residence | | 15 | 93.75% | All |  |  |  |  |  |
| 1. Average household | | 9 | 56.25% | FR  OEND  PRS |  |  |  |  |  |
| 1. Bedroom | | 8 | 50% | All |  |  |  |  |  |
| 1. Low socio-economic household | | 9 | 56.25% | All |  |  |  |  |  |
| **Body position** | |  |  |  | **Body position** | |  |  |  |
| 1. Contorted | | 7 | 43.75% | All |  |  |  |  |  |
| 1. Supine | | 9 | 56.25% | All |  |  |  |  |  |
| **Clothing** | |  |  |  | **Clothing** | |  |  |  |
| 1. Average | | 12 | 75% | All |  |  |  |  |  |
| **Physical characteristics** | |  |  |  | **Physical characteristics** | |  |  |  |
| 1. Skin | | 15 | 93.75% | All | 1. Older individual | | 7 | 50% | All |
| 1. Cyanotic | | 12 | 75% | All | 1. Skin | | 10 | 71.43% | All |
| 1. Diaphoretic | | 9 | 56.25% | ED  FR  OEND | 1. Pale | | 6 | 42.86% | All |
| 1. Pale | | 8 | 50% | ED  FR  OEND |  |  |  |  |  |
| 1. Injection lesions or abscesses | | 12 | 75% | All |  |  |  |  |  |
| **Breathing characteristics** | |  |  |  | **Breathing characteristics** | |  |  |  |
| 1. Before naloxone | | 15 | 93.75% | All | 1. Before naloxone | | 12 | 85.71% | All |
| 1. Decreased respiratory drive | | 13 | 81.25% | All | 1. Decreased respiratory drive | | 11 | 78.57% | All |
| 1. Sonorous | | 10 | 62.5% | All |  |  |  |  |  |
| 1. After naloxone | | 12 | 75% | All |  |  |  |  |  |
| 1. Increased respiratory rate | | 8 | 50% | ED  FR  OEND |  |  |  |  |  |
| **Persons’ responsiveness** | |  |  |  | **Persons’ responsiveness** | |  |  |  |
| 1. Before naloxone | | 16 | 100% | All | 1. Before naloxone | | 10 | 71.43% | ED  FR  PRS |
| 1. Decreased level of consciousness (LOC) | | 11 | 68.75% | All | 1. Decreased level of consciousness (LOC) | | 8 | 57.14% | ED  FR |
| 1. Unresponsive | | 13 | 81.25% | All | 1. Unresponsive | | 6 | 42.86% | ED  FR  PRS |
| 1. After naloxone | | 13 | 81.25% | All | 1. After naloxone | | 7 | 50% | FR  PRS |
| 1. Aggressive, agitated, or combative | | 11 | 68.75% | All |  |  |  |  |  |
| 1. Rapidly improve | | 7 | 43.75% | All |  |  |  |  |  |
| 1. Remain minimally responsive | | 7 | 43.75% | All |  |  |  |  |  |
| **Items near the person** | |  |  |  | **Items near the person** | |  |  |  |
| 1. Drug paraphernalia | | 15 | 93.75% | All | 1. Pill bottles | | 9 | 64.29% | All |
| 1. Containers with pills | | 9 | 56.25% | All |  |  |  |  |  |
| 1. Powder or residue | | 7 | 43.75% | FR  OEND  PRS |  |  |  |  |  |
| 1. Syringes | | 11 | 68.75% | All |  |  |  |  |  |
| 1. Ice, snow, or water on person | | 7 | 43.75% | All |  |  |  |  |  |
| **Bystanders** | |  |  |  | **Bystanders** | |  |  |  |
| 1. Affects | | 12 | 75% | All | 1. Affects | | 7 | 50% | ED  FR  OEND |
| 1. Distrust^a^ | | 9 | 56.25% | All | 1. Concerned | | 7 | 50% | All |
| 1. Fearful | | 12 | 75% | All | 1. Behaviors | | 12 | 85.71% | All |
| 1. Behaviors^b^ | | 16 | 100% | All | 1. Recommended rescue interventions | | 7 | 50% | All |
| 1. Call EMS | | 10 | 62.5% | All | 1. Type | | 12 | 85.71% | All |
| 1. Not recommended rescue interventions^c^ | | 11 | 68.75% | All | 1. Family and friends | | 12 | 85.71% | All |
| 1. Recommended rescue interventions | | 10 | 62.5% | All |  |  |  |  |  |
| 1. Type | | 15 | 93.75% | All |  |  |  |  |  |
| 1. Family and friends | | 13 | 81.25% | All |  |  |  |  |  |
| 1. Strangers | | 7 | 43.75% | ED  FR  OEND |  |  |  |  |  |
| **Administration of naloxone** | |  |  |  | **Administration of naloxone** | |  |  |  |
| 1. Dose | | 11 | 68.75% | All | 1. Naloxone not administered | | 6 | 42.86% | ED  FR  PRS |
| 1. Onset of response | | 9 | 56.25% | All |  |  |  |  |  |
| 1. Route | | 10 | 62.5% | All |  |  |  |  |  |
| 1. Side effects | | 9 | 56.25% | All |  |  |  |  |  |
|  |  |  |  |  | **Intentional overdose** | |  |  |  |
|  |  |  |  |  | 1. Suicide attempt | | 9 | 64.29% | ED  FR  OEND |
|  |  |  |  |  | **Unintentional overdose** | |  |  |  |
|  |  |  |  |  | 1. Mixed-up medications | | 8 | 57.14% | All |

ED; emergency department, FR; first responder, OEND: OEND instructor, PRS; peer recovery specialist

^a^ ‘Bystanders’ ‘Affects’ ‘Distrust’ include 1 transcript from the prescription opioids section because participants referenced illicit opioids.

^b^ ‘Bystanders’ ‘Behaviors’ include 2 transcripts from the prescription opioids section because participants referenced illicit opioids.

^c^ ‘Not recommended rescue interventions’ include 1 transcript from the prescription opioids section because participants referenced illicit opioids.
