# Supplementary material for: Developing medical simulations for opioid overdose response training: A qualitative analysis of narratives from responders to overdoses
Source: PLoS One. 2024 Mar 28;19(3):e0294626. doi: 10.1371/journal.pone.0294626 (PMC10977769; doi:10.1371/journal.pone.0294626)
Supplement: S2 Table — ED; emergency department, FR; first responder, OEND: OEND instructor, PRS; peer recovery specialist. a ‘Bystanders’ ‘Affects’ ‘Distrust’ include 1 transcript from the prescription opioids section because participants referenced illicit opioids. b ‘Bystanders’ ‘Behaviors’ include 2 transcripts from the prescription opioids section because participants referenced illicit opioids. c ‘Clean up drug paraphernalia’ include 1 transcript from the prescription opioids section because participants referenced illicit opioids. d ‘Not recommended rescue interventions’ include 1 transcript from the prescription opioids section because participants referenced illicit opioids. (DOCX) [file pone.0294626.s002.docx]

**S2 Table.** Themes and subthemes of the locations and physical characteristics of illicit and prescription opioid overdoses as described by participants and organized by coders

| **Illicit opioids** | | **# of transcripts** | **% of transcripts** | **Expert** | **Prescription opioids** | | **# of transcripts** | **% of transcripts** | **Expert** |
| --- | --- | --- | --- | --- | --- | --- | --- | --- | --- |
| **Location** | |  |  |  | **Location** | |  |  |  |
| 1. Abandoned building | | 1 | 6.25% | ED | 1. Grocery store | | 2 | 14.29% | FR |
| 1. Brought to ED by EMS | | 2 | 12.5% | ED | 1. Nursing home | | 4 | 28.57% | ED  FR |
| 1. Brought to ED by non-EMS | | 2 | 12.5% | ED | 1. Personal residence | | 11 | 78.57% | All |
| 1. Car | | 8 | 50% | ED  FR  OEND | 1. Bedroom | | 5 | 35.71% | FR  PRS |
| 1. Hotel or motel | | 8 | 50% | All | 1. Dining room | | 2 | 14.29% | FR |
| 1. Jail | | 1 | 6.25% | OEND | 1. High socio-economic household | | 1 | 7.14% | FR |
| 1. Public spaces | | 4 | 25% | ED  FR  OEND | 1. Low socio-economic household | | 1 | 7.14% | FR |
| 1. Personal residence | | 15 | 93.75% | All | 1. Trap house | | 1 | 7.14% | OEND |
| 1. Apartment | | 4 | 25% | FR  OEND  PRS | 1. Retirement community or assisted living | | 3 | 21.43% | ED  FR |
| 1. Average household | | 9 | 56.25% | FR  OEND  PRS |  |  |  |  |  |
| 1. Multi-generational home | | 1 | 6.25% | FR |  |  |  |  |  |
| 1. Bathroom | | 5 | 31.25% | All |  |  |  |  |  |
| 1. Bedroom | | 8 | 50% | All |  |  |  |  |  |
| 1. Cluttered | | 5 | 31.25% | ED  FR  OEND |  |  |  |  |  |
| 1. Living room | | 3 | 18.75% | OEND  PRS |  |  |  |  |  |
| 1. Low socio-economic household | | 9 | 56.25% | All |  |  |  |  |  |
| 1. Stranger’s house | | 1 | 6.25% | ED |  |  |  |  |  |
| 1. Trailer | | 4 | 25% | FR  OEND |  |  |  |  |  |
| 1. Trap house | | 2 | 12.5% | OEND |  |  |  |  |  |
| **Body position** | |  |  |  | **Body position** | |  |  |  |
| 1. Contorted | | 7 | 43.75% | All | 1. In bed | | 5 | 35.71% | FR  PRS |
| 1. Packed with ice | | 3 | 18.75% | ED  FR | 1. Laying on porch | | 1 | 7.14% | OEND |
| 1. Sitting | | 2 | 12.5% | OEND | 1. Sitting | | 2 | 14.29% | FR  OEND |
| 1. Supine | | 9 | 56.25% | All | 1. Supine | | 2 | 14.29% | FR |
| **Clothing** | |  |  |  | **Clothing** | |  |  |  |
| 1. Average | | 12 | 75% | All | 1. Dressed for bed | | 1 | 7.14% | FR |
| 1. Hard to classify to one type | | 2 | 12.5% | ED  FR | 1. Elderly clothing | | 1 | 7.14% | ED |
| 1. Naked | | 1 | 6.25% | ED | 1. No pattern to clothing | | 5 | 35.71% | ED  FR  OEND |
| 1. Not appropriate for season or sex | | 2 | 12.5% | FR  PRS | 1. Normal or well-kempt | | 5 | 35.71% | ED  FR  OEND |
| 1. Unkempt | | 3 | 18.75% | ED  FR | 1. Unkempt | | 1 | 7.14% | PRS |
| 1. Wet | | 5 | 31.25% | ED  FR  PRS |  |  |  |  |  |
| **Physical characteristics** | |  |  |  | **Physical characteristics** | |  |  |  |
| 1. Age | | 1 | 6.25% | ED | 1. Body temperature | | 1 | 7.14% | FR |
| 1. Nauseous | | 1 | 6.25% | ED | 1. Cellulitis | | 1 | 7.14% | OEND |
| 1. Pinpoint pupils | | 6 | 37.5% | ED  FR  OEND | 1. Dependent on situation | | 1 | 7.14% | FR |
| 1. Skin | | 15 | 93.75% | All | 1. Obese | | 1 | 7.14% | OEND |
| 1. Cyanotic | | 12 | 75% | All | 1. Older individual | | 7 | 50% | All |
| 1. Diaphoretic | | 9 | 56.25% | ED  FR  OEND | 1. Pupils | | 2 | 14.29% | ED |
| 1. Pale | | 8 | 50% | ED  FR  OEND | 1. Normal | | 1 | 7.14% | ED |
| 1. Piloerection | | 1 | 6.25% | ED | 1. Pinpoint | | 3 | 21.43% | ED  FR |
| 1. Injection lesions or abscesses | | 12 | 75% | All | 1. Skin | | 10 | 71.43% | All |
| 1. Thin | | 1 | 6.25% | ED | 1. Cyanotic | | 3 | 21.43% | FR  OEND  PRS |
|  |  |  |  |  | 1. Diaphoretic | | 2 | 14.29% | OEND  PRS |
|  |  |  |  |  | 1. No common characteristics | | 1 | 7.14% | ED |
|  |  |  |  |  | 1. Normal | | 1 | 7.14% | ED |
|  |  |  |  |  | 1. Pale | | 6 | 42.86% | All |
|  |  |  |  |  | 1. Younger individual | | 2 | 14.29% | FR  OEND |
| **Breathing characteristics** | |  |  |  | **Breathing characteristics** | |  |  |  |
| 1. Before naloxone | | 15 | 93.75% | All | 1. Before naloxone | | 12 | 85.71% | All |
| 1. Apneic (not breathing) | | 5 | 31.25% | ED  FR  OEND | 1. Decreased respiratory drive | | 11 | 78.57% | All |
| 1. Bag-valve-mask | | 4 | 25% | ED  FR | 1. Normal respiratory drive | | 3 | 21.43% | ED |
| 1. Decreased respiratory drive | | 13 | 81.25% | All | 1. Not breathing | | 4 | 28.57% | FR  OEND  PRS |
| 1. Place a nasopharyngeal or oropharyngeal airway | | 1 | 6.25% | ED | 1. Require intubation | | 1 | 7.14% | ED |
| 1. Sonorous | | 10 | 62.5% | All | 1. Sonorous | | 2 | 14.29% | PRS |
| 1. After naloxone | | 12 | 75% | All | 1. After naloxone | | 3 | 21.43% | FR  OEND  PRS |
| 1. Gasp for air | | 5 | 31.25% | ED  OEND  PRS | 1. Gasp for air | | 1 | 7.14% | PRS |
| 1. Increased respiratory rate | | 8 | 50% | ED  FR  OEND | 1. Increased respiratory drive | | 1 | 7.14% | FR |
| 1. No improvement after first dose | | 5 | 31.25% | ED  OEND | 1. No return of breathing | | 1 | 7.14% | OEND |
| **Heart characteristics** | |  |  |  | **Heart characteristics** | |  |  |  |
| 1. Bradycardic | | 3 | 18.75% | ED  OEND |  |  |  |  |  |
| 1. Tachycardic | | 4 | 25% | ED  FR  PRS |  |  |  |  |  |
| **Persons’ responsiveness** | |  |  |  | **Persons’ responsiveness** | |  |  |  |
| 1. Before naloxone | | 16 | 100% | All | 1. Before naloxone | | 10 | 71.43% | ED  FR  PRS |
| 1. Alert and high energy | | 1 | 6.25% | PRS | 1. Confused | | 2 | 14.29% | ED  FR |
| 1. Decreased level of consciousness (LOC) | | 11 | 68.75% | All | 1. Cooperative or communicating | | 2 | 14.29% | ED  FR |
| 1. Sluggish | | 6 | 37.5% | FR  OEND  PRS | 1. Decreased level of consciousness (LOC) | | 8 | 57.14% | ED  FR |
| 1. Unresponsive | | 13 | 81.25% | All | 1. Sluggish | | 2 | 14.29% | ED  FR |
| 1. Ventilation improved LOC | | 1 | 6.25% | ED | 1. Uncooperative | | 1 | 7.14% | FR |
| 1. After naloxone | | 13 | 81.25% | All | 1. Unresponsive | | 6 | 42.86% | ED  FR  PRS |
| 1. Aggressive, agitated, or combative | | 11 | 68.75% | All | 1. After naloxone | | 7 | 50% | FR  PRS |
| 1. Confused or frightened | | 5 | 31.25% | FR  OEND | 1. Confused | | 1 | 7.14% | PRS |
| 1. Cooperative or communicating | | 5 | 31.25% | ED  FR  OEND | 1. Cooperative or communicating | | 2 | 14.29% | FR  PRS |
| 1. Rapidly improve | | 7 | 43.75% | All | 1. Embarrassed | | 1 | 7.14% | PRS |
| 1. Remain minimally responsive | | 7 | 43.75% | All | 1. Imperceptible | | 2 | 14.29% | FR |
|  |  |  |  |  | 1. Regain consciousness | | 1 | 7.14% | FR |
|  |  |  |  |  | 1. Sluggish | | 2 | 14.29% | FR |
| **Items near the person** | |  |  |  | **Items near the person** | |  |  |  |
| 1. Cell phones | | 3 | 18.75% | FR  PRS | 1. Dependent on situation | | 1 | 7.14% | FR |
| 1. Clothes, clutter, etc. | | 3 | 18.75% | FR  PRS | 1. Drug paraphernalia | | 1 | 7.14% | PRS |
| 1. Drug paraphernalia | | 15 | 93.75% | All | 1. Alcohol | | 1 | 7.14% | ED |
| 1. Cigarettes and alcohol | | 3 | 18.75% | FR  PRS | 1. Crushed medications | | 1 | 7.14% | PRS |
| 1. Containers with pills | | 9 | 56.25% | All | 1. Fentanyl patches | | 1 | 7.14% | ED |
| 1. Cotton | | 4 | 25% | ED  FR  OEND | 1. Medication list | | 3 | 21.43% | FR |
| 1. Smoking pipe | | 1 | 6.25% | ED | 1. Normal nightstand items | | 2 | 14.29% | FR |
| 1. Lighter or matches | | 1 | 6.25% | ED | 1. Pill bottles | | 9 | 64.29% | All |
| 1. Powder or residue | | 7 | 43.75% | FR  OEND  PRS |  |  |  |  |  |
| 1. Spoons | | 4 | 25% | ED  FR  OEND |  |  |  |  |  |
| 1. Syringes | | 11 | 68.75% | All |  |  |  |  |  |
| 1. Tourniquets | | 3 | 18.75% | ED  FR  OEND |  |  |  |  |  |
| 1. Ice, snow, or water on person | | 7 | 43.75% | All |  |  |  |  |  |
| 1. Naloxone | | 3 | 18.75% | ED  FR |  |  |  |  |  |
| 1. Nothing | | 3 | 18.75% | ED  OEND |  |  |  |  |  |
| **Bystanders** | |  |  |  | **Bystanders** | |  |  |  |
| 1. Affects | | 12 | 75% | All | 1. Affects | | 7 | 50% | ED  FR  OEND |
| 1. Concerned | | 5 | 31.25% | ED  FR  OEND | 1. Angry | | 1 | 7.14% | OEND |
| 1. Cooperative | | 1 | 6.25% | FR | 1. Concerned | | 7 | 50% | All |
| 1. Desperation | | 2 | 12.5% | OEND  PRS | 1. Dramatic | | 1 | 7.14% | ED |
| 1. Distrust^a^ | | 9 | 56.25% | All | 1. Fearful | | 1 | 7.14% | PRS |
| 1. Fearful | | 12 | 75% | All | 1. Indifferent | | 1 | 7.14% | FR |
| 1. Behaviors^b^ | | 16 | 100% | All | 1. Supportive or helpful | | 3 | 21.43% | FR |
| 1. Call EMS | | 10 | 62.5% | All | 1. Behaviors | | 12 | 85.71% | All |
| 1. Clean up drug paraphernalia^c^ | | 4 | 25% | FR  OEND | 1. Call EMS | | 4 | 28.57% | ED  FR  PRS |
| 1. Come to hospital with person | | 1 | 6.25% | ED | 1. Clean up drug paraphernalia | | 2 | 14.29% | FR  PRS |
| 1. Flee the scene | | 5 | 31.25% | ED  OEND  PRS | 1. Come to hospital with person | | 4 | 28.57% | ED  FR  PRS |
| 1. Injured at scene | | 1 | 6.25% | OEND | 1. Compile person’s prescriptions | | 4 | 28.57% | ED  FR |
| 1. Not recommended rescue interventions^d^ | | 11 | 68.75% | All | 1. Lying | | 2 | 14.29% | FR  PRS |
| 1. Recommended rescue interventions | | 10 | 62.5% | All | 1. Not recommended rescue interventions | | 3 | 21.43% | FR  PRS |
| 1. Type | | 15 | 93.75% | All | 1. Recommended rescue interventions | | 7 | 50% | All |
| 1. Dealer | | 1 | 6.25% | FR | 1. Type | | 12 | 85.71% | All |
| 1. Family and friends | | 13 | 81.25% | All | 1. Family and friends | | 12 | 85.71% | All |
| 1. No bystanders present | | 4 | 25% | ED  FR  OEND | 1. Healthcare providers | | 3 | 21.43% | FR |
| 1. Strangers | | 7 | 43.75% | ED  FR  OEND | 1. Law enforcement officers | | 1 | 7.14% | OEND |
|  |  |  |  |  | 1. No bystanders present | | 3 | 21.43% | ED  FR |
|  |  |  |  |  | 1. Strangers | | 1 | 7.14% | FR |
| **Administration of naloxone** | |  |  |  | **Administration of naloxone** | |  |  |  |
| 1. Dose | | 11 | 68.75% | All | 1. Naloxone administered | | 4 | 28.57% | FR  OEND  PRS |
| 1. Duration | | 4 | 25% | ED  FR  OEND | 1. Naloxone not administered | | 6 | 42.86% | ED  FR  PRS |
| 1. No harm | | 2 | 12.5% | PRS | 1. Not in protocol | | 2 | 14.29% | ED  FR |
| 1. Onset of response | | 9 | 56.25% | All | 1. Not titrated | | 1 | 7.14% | FR |
| 1. Refusal of care | | 3 | 18.75% | All |  |  |  |  |  |
| 1. Route | | 10 | 62.5% | All |  |  |  |  |  |
| 1. Side effects | | 9 | 56.25% | All |  |  |  |  |  |
| 1. Titration | | 6 | 37.5% | ED  FR |  |  |  |  |  |
| 1. Treat respiratory depression, then consider naloxone | | 2 | 12.5% | ED  FR |  |  |  |  |  |
|  |  |  |  |  | **Intentional overdose** | |  |  |  |
|  |  |  |  |  | 1. Suicide attempt | | 9 | 64.29% | ED  FR  OEND |
|  |  |  |  |  | 1. Cannot refuse care | | 1 | 7.14% | FR |
|  |  |  |  |  | 1. Consumed family member’s medication | | 2 | 14.29% | FR  OEND |
|  |  |  |  |  | 1. Consumption of illicit medication | | 2 | 14.29% | ED  FR |
|  |  |  |  |  | 1. Consumption of prescribed medication | | 4 | 28.57% | ED  FR |
|  |  |  |  |  | 1. Depressed | | 3 | 21.43% | ED  FR |
|  |  |  |  |  | 1. Elderly individual | | 2 | 14.29% | FR |
|  |  |  |  |  | 1. Fentanyl patch | | 1 | 7.14% | ED |
|  |  |  |  |  | 1. Forthcoming about intentions | | 5 | 35.71% | ED  FR |
|  |  |  |  |  | 1. Polypharmacy | | 3 | 21.43% | ED  FR |
|  |  |  |  |  | 1. Younger individual | | 2 | 14.29% | FR |
|  |  |  |  |  | **Unintentional overdose** | |  |  |  |
|  |  |  |  |  | 1. Accept care | | 1 | 7.14% | FR |
|  |  |  |  |  | 1. Confused | | 1 | 7.14% | FR |
|  |  |  |  |  | 1. Mixed-up medications | | 8 | 57.14% | All |
|  |  |  |  |  | 1. Overprescribed or overmedicated | | 5 | 35.71% | ED  FR |

ED; emergency department, FR; first responder, OEND: OEND instructor, PRS; peer recovery specialist

^a^ ‘Bystanders’ ‘Affects’ ‘Distrust’ include 1 transcript from the prescription opioids section because participants referenced illicit opioids.

^b^ ‘Bystanders’ ‘Behaviors’ include 2 transcripts from the prescription opioids section because participants referenced illicit opioids.

^c^ ‘Clean up drug paraphernalia’ include 1 transcript from the prescription opioids section because participants referenced illicit opioids.

^d^ ‘Not recommended rescue interventions’ include 1 transcript from the prescription opioids section because participants referenced illicit opioids.
