# Supplementary material for: Developing medical simulations for opioid overdose response training: A qualitative analysis of narratives from responders to overdoses
Source: PLoS One. 2024 Mar 28;19(3):e0294626. doi: 10.1371/journal.pone.0294626 (PMC10977769; doi:10.1371/journal.pone.0294626)
Supplement: S3 Table — One ED physician did not complete the OOKS. (DOCX) [file pone.0294626.s003.docx]

**S3 Table**. Average opioid overdose knowledge scale (OOKS) scores among participants

|  | ED physicians (n=4) | First responders (n=5) | OEND instructors (n=5) | Peer recovery specialists (n=2) | **All participants (n=16)** |
| --- | --- | --- | --- | --- | --- |
| **OOKS, mean (SD)** | 42.00 (1.41) | 39.40 (3.21) | 41.60 (2.51) | 40.00 (2.83) | **40.81 (2.59)** |
| Risk, mean (SD) | 8.00 (1.15) | 6.20 (1.10) | 8.60 (0.55) | 7.50 (2.12) | **7.56 (1.41)** |
| Signs, mean (SD) | 9.50 (1.00) | 9.00 (1.41) | 8.40 (0.55) | 8.00 (2.83) | **8.81 (1.28)** |
| Action, mean (SD) | 9.25 (1.50) | 10.20 (1.30) | 10.40 (0.55) | 10.50 (0.71) | **10.06 (1.12)** |
| Naloxone use, mean (SD) | 15.25 (1.26) | 14.00 (0.71) | 14.20 (2.17) | 14.00 (1.41) | **14.38 (1.45)** |

One ED physician did not complete the OOKS.
