# Supplementary material for: Developing medical simulations for opioid overdose response training: A qualitative analysis of narratives from responders to overdoses
Source: PLoS One. 2024 Mar 28;19(3):e0294626. doi: 10.1371/journal.pone.0294626 (PMC10977769; doi:10.1371/journal.pone.0294626)
Supplement: S4 Table — One ED physician did not complete the OOAS. (DOCX) [file pone.0294626.s004.docx]

**S4 Table**. Average opioid overdose attitude scale (OOAS) scores among participants

|  | ED physicians (n=4) | First responders (n=5) | OEND instructors (n=5) | Peer recovery specialists (n=2) | **All participants (n=16)** |
| --- | --- | --- | --- | --- | --- |
| **OOAS, mean (SD)** | 115.75 (3.30) | 114.40 (5.27) | 119.40 (4.56) | 109.50 (13.44) | **115.69 (6.12)** |
| Competence, mean (SD) | 48.25 (2.22) | 47.80 (2.17) | 49.40 (0.89) | 41.00 (7.07) | **47.56 (3.58)** |
| Concerns, mean (SD) | 35.25 (2.50) | 37.00 (4.06) | 37.80 (3.35) | 35.50 (6.36) | **36.63 (3.54)** |
| Readiness, mean (SD) | 32.25 (2.87) | 29.60 (1.82) | 32.20 (3.11) | 33.00 (0.00) | **31.50 (2.63)** |

One ED physician did not complete the OOAS.
