# Supplementary material for: Developing medical simulations for opioid overdose response training: A qualitative analysis of narratives from responders to overdoses
Source: PLoS One. 2024 Mar 28;19(3):e0294626. doi: 10.1371/journal.pone.0294626 (PMC10977769; doi:10.1371/journal.pone.0294626)
Supplement: S5 Table — (DOCX) [file pone.0294626.s005.docx]

**S5 Table.** Four medical simulations for out-of-hospital opioid overdoses including examples of illicit and prescription opioids

| **Illicit opioid overdose – Example 1** |
| --- |
| Location:   - Car |
| Person’s presentation:   - Slumped in car - Pale, cyanotic, diaphoretic - Injection lesions in antecubital fossa - Pinpoint pupils |
| Person’s symptoms/vitals:   - Unresponsive - Not breathing - Pulseless |
| Items near the person:   - Syringe (28-to-30-gauge) - Cooker with white, gray, tan residue |
| Presence of bystanders:   - None |
| Treatment approach:   - Primary treatment: Ventilations and chest compressions (defibrillation if indicated) - Secondary treatment: One to two doses of naloxone (0.4 mg to 4 mg per mL) |
| **Illicit opioid overdose – Example 2** |
| Location:   - Personal residence |
| Person’s presentation:   - Supine in bed - Pale, cyanotic, diaphoretic - Injection lesions in antecubital fossa - Pinpoint pupils |
| Person’s symptoms/vitals:   - Decreased LOC (GCS 8 to 10) - Decreased respiratory drive (6 to 8 breaths per minute) - Weak pulse |
| Items near the person:   - Syringe (28-to-30-gauge) - Cooker with white, gray, tan residue |
| Presence of bystanders:   - Friend: says they “came in and found them like this” - Affect: Distrust - Behavior: Alerted EMS, placed ice in the person’s pants, cleaned up drug paraphernalia |
| Treatment approach:   - Primary treatment: Two doses of naloxone (0.4 mg to 4 mg per mL) and ventilations |
| **Prescription opioid overdose – Example 1** |
| Location:   - Personal residence |
| Person’s presentation:   - Older individual - Supine in bed - Pale |
| Person’s symptoms/vitals:   - Decreased LOC (GCS 8 to 10) - Decreased respiratory drive (10 to 12 breaths per minute) |
| Items near the person:   - Pill bottles |
| Presence of bystanders:   - Family member who believes the person mixed-up their medications - Affect: Concerned - Behavior: Alerted EMS |
| Treatment approach:   - Primary treatment dependent on person’s responsiveness |
| **Prescription opioid overdose – Example 2** |
| Location:   - Personal residence |
| Person’s presentation:   - Older individual - Supine in bed - Pale |
| Person’s symptoms/vitals:   - Unresponsive - Decreased respiratory drive (6 to 8 breaths per minute) - Weak pulse |
| Items near the person:   - Pill bottles |
| Presence of bystanders:   - Family member who believes the person attempted suicide - Affect: Concerned - Behavior: Alerted EMS |
| Treatment approach:   - Primary treatment: Two doses of naloxone (0.4 mg to 4 mg per mL) |
