# Supplementary material for: Developing medical simulations for opioid overdose response training: A qualitative analysis of narratives from responders to overdoses
Source: PLoS One. 2024 Mar 28;19(3):e0294626. doi: 10.1371/journal.pone.0294626 (PMC10977769; doi:10.1371/journal.pone.0294626)
Supplement: S1 File — (DOCX) [file pone.0294626.s006.docx]

**Table 3.** Opioid overdose simulation scenario descriptions in the current literature

McDermott, C., & Collins, N. C. (2012). Prehospital medication administration: a randomised study comparing intranasal and intravenous routes. Emergency medicine international, 2012. doi: 10.1155/2012/476161

Edwards, E. T., Edwards, E. S., Davis, E., Mulcare, M., Wiklund, M., & Kelley, G. (2015). Comparative usability study of a novel auto-injector and an intranasal system for naloxone delivery. Pain and therapy, 4, 89-105. doi: 10.1007/s40122-015-0035-9

Kim, H. K., Connors, N. J., & Mazer-Amirshahi, M. E. (2019). The role of take-home naloxone in the epidemic of opioid overdose involving illicitly manufactured fentanyl and its analogs. Expert opinion on drug safety, 18(6), 465-475. doi: 10.1080/14740338.2019.1613372

Krieter, P., Chiang, N., Gyaw, S., Skolnick, P., Crystal, R., Keegan, F., Aker, J., Beck, M., & Harris, J. (2016). Pharmacokinetic properties and human use characteristics of an FDA‐approved intranasal naloxone product for the treatment of opioid overdose. The Journal of Clinical Pharmacology, 56(10), 1243-1253. doi: 10.1002/jcph.759

Kobayashi, L., Green, T. C., Bowman, S. E., Ray, M. C., McKenzie, M. S., & Rich, J. D. (2017). Patient simulation for assessment of layperson management of opioid overdose with intranasal naloxone in a recently released prisoner cohort. Simulation in healthcare, 12(1), 22-27. doi: 10.1097/SIH.0000000000000182

Eggleston, W., Podolak, C., Sullivan, R. W., Pacelli, L., Keenan, M., & Wojcik, S. (2018). A randomized usability assessment of simulated naloxone administration by community members. Addiction, 113(12), 2300-2304. doi: 10.1111/add.14416

Goldberg, S. A., Dworkis, D. A., Liao, V. T., Eyre, A. J., Albert, J., Fawcett, M. M., Narovec, C. M., DiClemente, J., & Weiner, S. G. (2018). Feasibility of bystander administration of public-access naloxone for opioid overdose. Prehospital Emergency Care, 22(6), 788-794. doi: 10.1080/10903127.2018.1461284

Eggleston, W., Calleo, V., Kim, M., & Wojcik, S. (2020). Naloxone administration by untrained community members. Pharmacotherapy: The Journal of Human Pharmacology and Drug Therapy, 40(1), 84-88. doi: 10.1002/phar.2352

Franko II, T. S., Distefano, D., & Lewis, L. (2019). A novel naloxone training compared with current recommended training in an overdose simulation. Journal of the American Pharmacists Association, 59(3), 375-378. doi: 10.1016/j.japh.2018.12.022
